# Wireless Sensor Networks for Heritage Object Deformation Detection and Tracking Algorithm

**DOI:** 10.3390/s141120562

**Published:** 2014-10-31

**Authors:** Zhijun Xie, Guangyan Huang, Roozbeh Zarei, Jing He, Yanchun Zhang, Hongwu Ye

**Affiliations:** 1 Department of Information Science and Engineering, Ningbo University, Ningbo 315021, China;; 2 School of Information Technology, Deakin University, Melbourne 3125, Australia; E-Mail: abysshuang@gmail.com; 3 College of Engineering and Science, Victoria University, Melbourne 3011, Australia; E-Mails: roozbeh.zarei@live.vu.edu.au (R.Z.); Jing.He@vu.edu.au (J.H.); Yanchun.Zhang@vu.edu.au (Y.Z.); 4 Zhejiang Fashion Institute of Technology, P. R. 315021, China; E-Mail: yhw@zjff.edu.cn

**Keywords:** sensor networks, heritage object monitoring, deformation, detection and tracking

## Abstract

Deformation is the direct cause of heritage object collapse. It is significant to monitor and signal the early warnings of the deformation of heritage objects. However, traditional heritage object monitoring methods only roughly monitor a simple-shaped heritage object as a whole, but cannot monitor complicated heritage objects, which may have a large number of surfaces inside and outside. Wireless sensor networks, comprising many small-sized, low-cost, low-power intelligent sensor nodes, are more useful to detect the deformation of every small part of the heritage objects. Wireless sensor networks need an effective mechanism to reduce both the communication costs and energy consumption in order to monitor the heritage objects in real time. In this paper, we provide an effective heritage object deformation detection and tracking method using wireless sensor networks (EffeHDDT). In EffeHDDT, we discover a connected core set of sensor nodes to reduce the communication cost for transmitting and collecting the data of the sensor networks. Particularly, we propose a heritage object boundary detecting and tracking mechanism. Both theoretical analysis and experimental results demonstrate that our EffeHDDT method outperforms the existing methods in terms of network traffic and the precision of the deformation detection.

## 1. Introduction

A culture heritage site is often an invaluable historical legacy. Different from the stone ruins in Europe, many of the heritage sites in Asia (e.g., China) are often damaged due in some part to natural-deformation-caused collapse, since they are built using clay and have complicated structures that are composed of a large number of surfaces inside and outside or that are arranged in a very long, zigzag way; typical examples include the ancient Great Wall, the Xi'an imperial city wall ruins of the Sui and Tang Dynasties, the Terracotta Army, the Yang Mausoleum of the Han Dynasty and the Dunhuang Mogao Grottoes. Deformation, which causes the split, collapse and destruction of parts of or whole heritage sites, is mainly responsible for heritage site damage. Therefore, it is significant to monitor and signal the early warnings of the deformation of heritage objects.

The existing monitoring methods [1–9] are not sustainable for the surveillance of heritage clay sites [2–7], since they only roughly monitor a simple heritage object as a whole, but cannot monitor heritage objects with complicated structures (*i.e.*, with a large number of surfaces inside and outside [8,9]). Although, a wireless sensor network was applied in the protection of heritage objects for its characteristics of easy deployment and extendability. For example, in recent years, researchers have deployed sensor networks at clay sites, but only for environmental status monitoring, such as collecting data of temperature and humidity [10–13].

Most of the existing work on localization and tracking using wireless sensor networks focuses specifically on the tracking of individual targets (e.g., people, animals and vehicles), such as CTBD (cooperative tracking with binary-detection) [14], DCTC (dynamic convoy tree-based collaboration) [15], DPR (dual prediction-based reporting) [16], unscented Kalman filter [17], the DCS (dynamic clustering scheme) algorithm [18], CODA (continuous object detection and tracking algorithm) [19], etc. However, detecting and tracking the deformation of a heritage site as a whole object faces new challenges. First, we should deploy a network of wireless sensor nodes to a large area or a complicated structure for monitoring every small part of the heritage site. Furthermore, the wireless sensor network should continuously monitor the site online for several years to capture the slow deformation caused often by natural forces.

In this paper, we propose an effective heritage detection and tracking (EffeHDDT) method to tackle these challenges. The EffeHDDT method mainly is comprised of the following two phases. In the initialization phase, EffeHDDT deploys the sensor nodes and anchor nodes, constructs a set of connected core sensor nodes and determines the initial boundary of the heritage object; the connected core includes a small number of domain heads and gateway nodes. Then, the control message is transmitted through the connected core to minimize the communication cost. In the monitoring phase, the EffeHDDT measures those tiny slow deformations of precious and small heritage sites by detecting the changing of the anchor node's RSSI (Receive Signal Strength Indication) value periodically. As for those large heritage objects established in a wild, relatively poor environment, the EffeHDDT detects and tracks the boundary of the heritage object periodically. We detect the deformation and collapse of the heritage object through checking whether a part of or the whole heritage object boundary moves out of the sensing range of the current boundary sensors; note that the membership of the heritage object boundary node set must be updated to be responsible for a new boundary location.

The advantage of EffeHDDT is that the whole network was divided into domains, and the sensors in the domain are all neighbors, which improves the accuracy of the boundary detection; meanwhile, the connected core reduces the traffic cost for sending the control message to the sink. Another advantage is that the EffeHDDT method enables each sensor node to detect and track the static or moving boundaries of heritage objects in the sensing field, taking advantage of finding boundary sensors (FBS) and achieves greater boundary estimation precision irrespective of the size of the heritage sites and the sensor network density.

The work most related is the DCS (dynamic clustering scheme) algorithm [18] and CODA (continuous object detection and tracking algorithm) [19]; both of them explored the feasibility of using WSNs to detect and track continuous objects. Our EffeHDDT method overcomes the above-mentioned two limitations of DCS and CODA by reducing the communication cost in two corresponding aspects: (1) the domain head in EffeHDDT can find the boundary sensors by the information of the sensors within the domain, so the cost for finding boundary sensors is changed from global communications to local communications; (2) EffeHDDT has already established a back-bone-like core set to efficiently pass messages when the sensor networks are deployed and, thus, does not incur communication costs for the reconstruction of clusters. In addition, EffeHDDT can detect and track the deformation of large-scale or complicated heritage objects by detecting the changing of or the movement of the heritage object boundary. An experimental study shows that our EffeHDDT outperforms DCS and CODA to detect and track heritage deformation, both expending less energy and having greater precision.

The rest of this paper is organized as follows. Section 2 briefly overviews related work. Section 3 details the EffeHDDT method. Section 4 conducts an experimental study to validate the effectiveness of the proposed method. Section 5 concludes the paper.

## 2. Related Works

In this Section, we conduct a survey of the existing heritage site protection methods and the sensor networks that are applied for monitoring the culture heritage objects and object tracking technology of sensor networks.

The existing heritage site protection methods are mainly used for four applications: the reinforcement and consolidation of the ancient heritage sites [2,3], the diagnosing of the main diseases of the heritage sites and their cause analysis [4,5], the weathering mechanism and protection of the ancient sites [6,7] and the study of the investigation and monitoring of earthen sites [8,9].

In recent years, sensor networks have become popular to monitor cultural heritage objects. Casciati *et al.* at Pavia University in Italy proposed a wireless sensor network technology to protect cultural heritage in Italy in 2004 [10]. The researchers at Trento University deployed a WSN monitoring system in Torre Aquila tower to monitor its structure and environment. The researchers at Madeira University of Portugal developed a WSN at the Fortaleza Sho Tiago Museum for monitoring and protecting the art environment. Jongwoo Sung from South Korean deployed a sensor network around the temple for forest fire detection [20]. The researchers from the Institute of Computing Technology, Chinese Academy of Sciences, deployed a sensor network to monitor the imperial palace cultural relics exhibition hall [10–13]. However, these applications simply collected the site environmental data, but there is no study on detecting and tracking the deformation of heritage sites. Fu *et al.* [21] proposed the judgment of the heritage object deformation method based on the cloud model. However, the heritage object deformation method based on the cloud model did not define deformation accuracy nor did it predict the deformation trends.

The WSN object tracking techniques can be classed into five groups: the binary detection-based tracking method [14,22,23], the delivery tree-based tracking method [15,24], the prediction-based tracking method [16,25–27], the particle filter-based tacking method [17,28–31] and the cluster-based tracking method [18,19,32–34]. However, these schemes focus only on the tracking of individual targets, e.g., people, animals, vehicles, and so forth. These methods are unsuitable for heritage object deformation detection and tracking, due to the requirements of large amounts of computation and communication. The cluster-based tracking methods are most related to this paper, such as the cluster-based distributed object tracking algorithm [32], DCS [18], CODA [19], Voronoi-based cluster tracking [33] and DCR [34]. After detecting the target, the nodes calculate the weight according to the motion state and the distance between the node and objects. The nodes can track the object only if the node's weight was higher than the set threshold. In DCS, the sensors automatically declare themselves as boundary sensors when they detect the presence of the object. All of these boundary sensors then were constructed into a group of dynamic clusters. A head of the cluster fuses the local boundary information and transmits this information to the sink. Once the sink has received boundary information from all of the dynamic clusters, it estimates the global boundary of the target object. While in CODA, a hybrid static/dynamic clustering technique was proposed to detect and track continuous objects: the sensors detect the continuous objects in the static cluster and send the local boundary information to the static cluster head to fuse firstly; then, the cluster head forms these boundary sensors into a dynamic cluster; it then sends the boundary information of this dynamic cluster to sinks. However, both DCS and CODA incur an expensive communication cost for object boundary detection and tracking. First, the boundary sensors in DCS and CODA are identified by requiring all sensors that detect the emergence of the object to communicate with all of their one-hop neighbors in the whole network to confirm that they detect the same object, which requires a significant communication cost and has a very high number of message exchanges. Second, the DCS and CODA require significant additional communication overheads to reconstruct dynamic clusters when the monitored object increases in size, changes in shape or moves over time.

Our EffeHDDT method overcomes the limitations of existing communication-consuming sensor networks and focuses on detecting the deformation of the heritage objects and continuously tracking the deformation changing with time.

## 3. Effective Heritage Deformation Detection and Tracking (EffeHDDT) Algorithm

We first provide an overview of EffeHDDT in Section 3.1. Then, Section 3.2 details the algorithm of the automatic construction of the connected core (ACCC). Section 3.3 studies the methods of boundary detection and the boundary tracking.

### 3.1. Overview of EffeHDDT

We develop the EffeHDDT method as shown in Algorithm 1 based on the domain and connected core presented in Section 3.2.


**Algorithm 1** The EffeHDDT Method
**Step 1:** Constructing the connected core and dividing the domain of the network by executing ACCC algorithm.**Step 2:** determines the initial boundary of heritage site.**Step 3:** detects and tracks the deformation of the heritage periodically.


The main idea of EffeHDDT is as follows. In Step 1, the EffeHDDT is divided into the initial stage and the monitoring stage. In the initialization phase, the EffeHDDT constructs the connected core and determines the initial boundary of the heritage object and deploys the anchor nodes on the heritage object; while in the monitoring phase, the EffeHDDT detects and tracks the deformation of the heritage object periodically. Then, after the sensor networks have been deployed at the heritage site, the EffeHDDT forms the domain head and gateway by executing the automatic construction of the connected core (ACCC) algorithm illustrated in Section 3.2 and by dividing the domain of the network. In Step 2, for those precious and small heritage objects, such as a Buddha statue, stored in a museum or a temple, where the environment is better than outdoors, the deformation is very small. We measure these tiny slow deformations by detecting the changing of the node's RSSI value periodically.

However, some large heritage objects, such as Chinese ancient Great Wall and Mount Li Buddha, are established in a wild, relatively poor environment and, thus, are vulnerable to earthquakes, landslides and other disasters that may induce sudden and greater collapse. If the deformation is not detected in a timely manner and treated, the partially collapse of the heritage object will lead to large-scale collapse and even the whole collapse of the heritage object. We measure these large-scale and sudden deformations by detecting the change of the heritage's boundary. If the heritage site is located within a single domain, the domain head detects and initializes the boundary of the heritage site by executing the FBS (finding boundary sensors algorithm in Section 3.1); otherwise, if the heritage site crosses multiple domains, the domain head estimates the portion of the boundary lying within its own domain, fuses the boundary information in a compact data format and then relays it to the sink via the connected core. The sink determines the entire boundary of the heritage site by compiling the integrated boundary information received from all of the domain heads in the network. In Step 3, when a heritage site is deformed and a portion of or the whole heritage object boundary moves out of the sensing range of the current boundary sensors, the membership of heritage object boundary node set must be updated to be responsible for the new boundary location. The domain head identifies the nodes within its domain that detect the new boundary sensors of the heritage object by executing the boundary detection method in Section 3.3, and the heritage object boundary node set must be updated to be responsible for the new boundary location.

### 3.2. Construction of the Connected Core

#### 3.2.1. Algorithm of the Automatic Construction of the Connected Core (ACCC)

We assume that all of the nodes in the sensor networks can communicate effectively within the same communication range. Two nodes are adjacent if they are within the communication range of each other; actually, an adjacent link between two nodes is symmetrical. Therefore, the topology of a sensor network can be a simple connected undirected graph *G* = (*V, E*), where *V* is the set of vertices constituted by all nodes and *E* is the edge sets of all links. Based on these assumptions, we define the following three concepts as a basis to present our ACCC algorithm.

Definition 1 (core): Given a graph *G* = (*V, E*), where *V* is the vertices' sets of sensor nodes and *E* is the edge sets of all links, the node set *C* of *G* = (*C*⊆*V*) is a core if and only if *C* satisfies that for any node *p* in *V, p* either in *C* or *p* is a neighbor of the node *q* in *C*.

**Figure 1.**
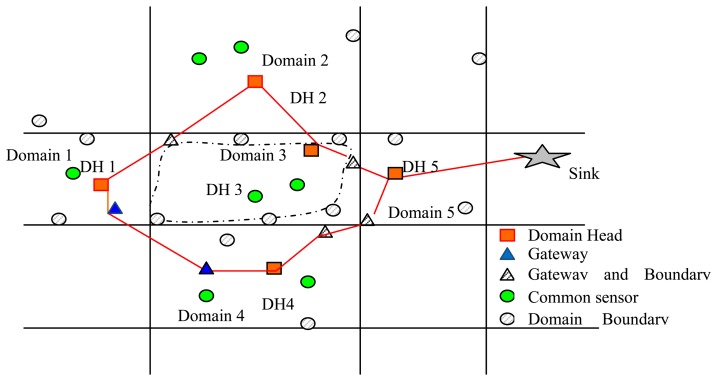
The connected core of sensor networks.

Definition 2 (connected core): Given a graph *G* = (*V, E*), the node set *C* of the graph G = (*C* ⊆*V*) is a connected core only if *C* satisfies the following conditions: The subgraph derived from *C* is a connected graph, and *C* is a key node set of graph *G*. For example, the connected key node set is constructed by the cluster head (with red squares) and the gateway (blue hollow triangle or practice triangle) of the sensor networks in Figure 1. The practice triangles in Figure 1 denote both the gateway and domain boundary sensors (Section 3.3). The practice dots represent the domain-boundary-sensors (Section 3.3), and the green hollow dots are the ordinary sensor nodes.

Definition 3 (domain): For the random sensor node *p*, we assume that the coordinate of *p* relative to the sink node *S* is *(x, y)*, sink node *S*, and all of the sensor nodes have the same communication radius *r*, then *p* belongs to the domain *(m, n)* if and only if the following equation holds: $m=\left[x/\frac{r}{\sqrt{2}}\right]$, $n=\left[y/\frac{r}{\sqrt{2}}\right]$. Where “/” denotes the division operator; “[]” is the integer operators for taking a valuegreater than or equal to an integer.

For example, if *r* = 15, the geographic coordinates of Node *A* are (40, 40); then *A_x_* = 4, *A_y_* = 4. Therefore, *A* belongs to the domain (4,4). We assume that the geographic coordinates of *B* are (51, 53), *B_x_* = 5, *B_y_* = 5, so *B* belongs to the domain (5,5), instead of (4,4).

For any sensor node *p*, we assume that the geographical coordinates are *(x, y)*. All of the nodes, including the sink node *S*, have the same communication range with a radius of *r*. For any sensor node *p*, we can calculate their respective domain according to Definition 3 and give the following ACCC algorithm. We required that the ACCC must be operated in the connectivity core *C* given in Definition 3:

**Algorithm 2** ACCC
**Step 1:** Every node *p* uses the GPS to calculate the geographic coordinates and the remaining energy; calculate the domain according to Definition 3.**Step 2:** Every node *p* periodically exchanges the comprehensive state information with its adjacent nodes.**Step 3:** Set the domain head and/or the domain gateway.**Step 4:** When the nodes get the sensing data, the data will be delivered directly to the domain head of the domain.


We explain the ACCC Algorithm 2 as follows. In Step 2, every node *p* periodically exchanges the comprehensive state information with its adjacent nodes. The comprehensive state information comprises three parameters:

(i) *S_p_*: the current status of node *p* (every node has three kinds of statuses: domain head, gateway or ordinary member);
(ii) *E_p_*: the remaining energy of *p*;
(iii) *G_p_*: the domain of *p*;
(iv) *(x,y)*: the geographical coordinates of *p*. After this operation, each node can get the adjacent nodes' information, such as the status of their neighbor nodes, the residual energy, their domain, the straight-line distance to the neighbor node and others.

In Step 3, initially, the status of the sink node is the domain head, while the others are members. In every cycle, node *p* will calculate its new status according to the following rules by its neighbor's information, such as the status *S_p_*, the energy *E_p_* and *G_p_*. If there is no domain head in the *G_p_*, the node with the largest residual energy in the *G_p_* will be selected as the domain head of this domain. Otherwise, if *p* is neither a domain head and gateway nor the neighborhood node of other domain, *p* is the gateway. In Step 4, when the nodes get the sensing data, the data will be delivered directly to the domain head of the domain.

#### 3.2.2. Connectivity Analysis

**Figure 2.**
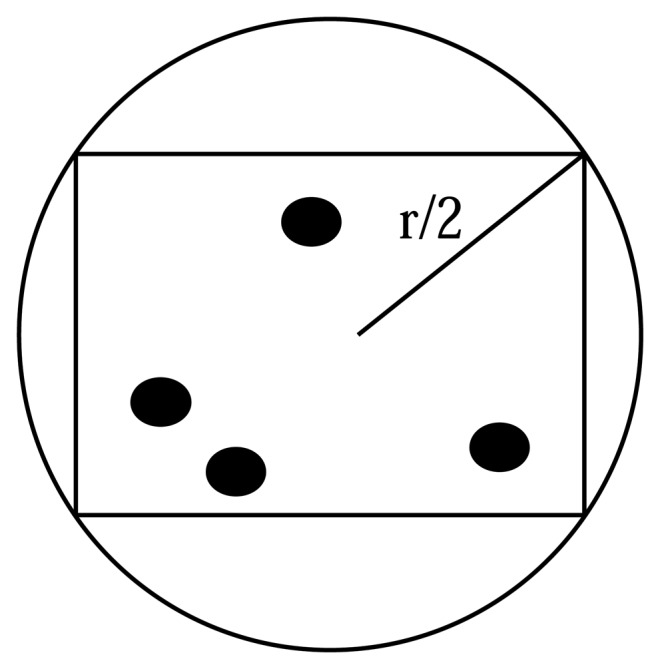
The inscribed square of the circle.

We can deduce Theorems 1 and 2 by Definition 3:

Theorem 1: Assume the sink node *S* and all sensor nodes in the sensor network have the same communication radius *r*; there is the unique division of domain that satisfied the formula in Definition 3.
Theorem 2: Any two sensor nodes in the same domain are adjacent nodes.
  Proof: According to Definition 3, the domain is a district of square, and the maximum distance between any two nodes of the region is $\sqrt{{\left(\frac{r}{\sqrt{2}}\right)}^{2}+{\left(\frac{r}{\sqrt{2}}\right)}^{2}}=r$. Any two nodes are within the radius of each other's effective communication, so any two sensor nodes in the same domain are adjacent nodes.
  As shown in Figure 2, let *r* be the communication radius of the sensor node; the domain is actually an inscribed square of the circle whose radius is *r*/2. The distance of any two points in the inscribed square is less than *r*, so that all of the nodes within the inscribed square can communicate with each other; thus, any two nodes within an inscribed square are connected.
  According to the algorithm ACCC we know that ACCC is fully distributed.
Theorem 3 Assuming the graph *G* = *(V, E)* is a simple, connected, undirected graph, the node set *ψ* = {*p* | *p* is the domain head or gateway node and *p* ∈ *V*} that is obtained from the algorithm ACCC is a connected core of graph *G*.
  Proof: First of all, we can see from Theorem 2 and Step 3 of the algorithm ACCC that every node in the set *ψ* is either a domain head node or its neighbor node is one of the domain heads at least; namely, it is a neighbor with some domain head node. Therefore, the set *ψ* is a core of the graph *G*. We prove that *ψ* is connected by induction as follows.
  Let *p* and *q* be any two domain head nodes of *ψ*, that is to say, *p,q*∈ *ψ*. We have assumed that all of the sensor nodes have the same communication radius, and for convenience of expression, we assume that the communication radius is one unit length; so, the distance between *p* and *q* is the length of the shortest path between *p* and *q*, denoted as *d(p,q)*. Since the graph Gis connected, *d(p,q)*<*R* (*R* is a real number), and *d(p,q)* is a finite integer.

(1) (i) If *d(p,q)* = 1, we can deduce that p and q are adjacent, so they are directly reachable. We can deduce that *ψ* is connected.
  (ii) If *d(p,q)* = 2, namely there is a path *(p, r, q)* in *G*, because of *d(p, q)* = 2; therefore, *p* and *q* are not adjacent. From Theorem 2, we know that *p* and *q* do not belong to the same domain. Because *r* is the neighbor of *p* and *q*, from Step 3.2 of the ACCC algorithm, if *r* is not the domain head, then *r* must be the gateway; therefore, *r* ∈ *ψ*. We can deduce that *ψ* is connected. (iii) If *d(p,q)* = 3, namely there is a path (*p,r*_1_,*r*_2_,*q)* in *G*. For the reason of *d(p,r*_2_*)* = 2, *p* and *q* are not adjacent, and we know that *p* and *r*_2_ do not belong to the same domain from Theorem 2. From Step 3.2 of the ACCC algorithm, we know that if *r*_1_ is not a domain head, *r*_1_ must be the gateway, and *r*_1_ is the neighbor of *p* and *r*_2_; so *r*_1_ ∈ *ψ*. For the same reason, since *d(r*_1_,*q)* = 2, we know that *r*_2_ ∈ *ψ*. We can deduce that *ψ* is connected.
(2) Assume that *ψ* is connected when *d(p,q)* = *m*(*m* > 3).
(3) For *d(p,q)* = *m* + *1*, there is a path *(p,r*_1_,*r*_2_,…,*r_m_,q)* in *G*. *ψ* is the core of graph *G*, and we can deduce that *r*_2_ is the domain head or *r*_2_ are neighbors of a domain head *r* in *ψ*. We can deduce d*(p,r)* ≤ 3. The proof of Step 1 shows that node *p* and *r* are reachable in *ψ*, but *d(r,q)* ≤ *m*; the induction assumption shows that node *p* and *r* are reachable within *ψ*. We can deduce *ψ* is connected.

From Theorem 3, we know that the nodes of set *ψ* build a connected core, and the connected core *ψ* is constituted of domain head nodes and gateway nodes. The Skyline query message and resulting data can be forwarded along the nodes in *ψ*. Compared to the entire network, the number of nodes in the *ψ* is much less, which can dramatically reduce network traffic.

### 3.3. Boundary Detection and Tracking

The key point for detection and tracking of deformation is to construct a set of connected core nodes in the sensor networks during the initial phase. As shown in Figure 1, one node within each domain is nominated as the head and plays the role of a local controller. The normal nodes get the sensor data of the environment and send or relay the sensing data to the domain head (DH). The DH generates sensing data of its own, collects the data sent from the normal nodes in the domain and fuses and transfers this information to the sink via the connected core path.

**Figure 3.**
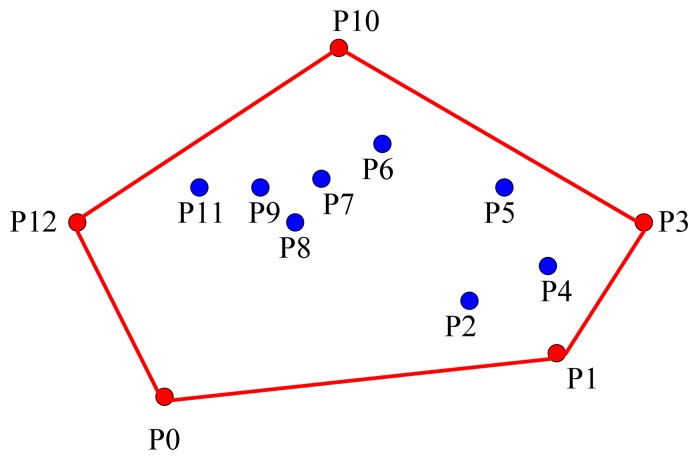
The minimum convex polygon.

When the DH receives the location information from all of the normal nodes in the domain, the DH detects the sensors located around at the boundary of the heritage object and notifies them that they are the boundary sensors of the heritage object. The boundary sensors are selected from the normal nodes in a domain through finding the minimum convex polygon that contains the heritage object. For a subset *S* of *n*-dimensional space *R, t* convex MCP(*K*) is defined as the smallest convex set in *R*. For example, the convex polygon represented by the red line shown in Figure 3 is the minimum convex polygon of convex set *Q* = *{p*_0_,*p*_1_…*p*_12_*}*. We present the algorithm for finding boundary sensors in Section 3.3.1; the method for finding the minimum convex polygon in geometry refers to [35].

#### 3.3.1. Finding Boundary Sensors (FBS) Algorithm

Let all of the nodes in a domain represent the subset *S* of n-dimensional space *R*, and the DH (domain head) distinguishes the boundary sensors among them by using the finding boundary sensors (FBS) algorithm in Algorithm 3.


**Algorithm 3** Finding the boundary sensors (FBS) algorithm
1: Input a set of sensors *S* = *{p*_0_,*p*_2_…*P_n_*_−1_*}*2: Select the rightmost and lowest sensor *p*_0_ as the original and establish a coordinate axis whose origin is *p*_0_.3: Map the other sensor *S* into the *p*_0_ origin coordinate axis system.4: Compute the slope of the sensor *S*.5: Let *T*[*n*] be the sorted array *S* in ascending order.6: Push *T*[*n* − 1] and *p*_0_ onto a stack *ST*, and *sp* denotes the stack point of *ST*.7: **WHILE** i<n.8:   **IF**
*D(ST*[*sp*],*ST*[*sp* − 1],*T*[*i*]*)*≥*0*, **THEN**9:       Push *T*[*i*] into *ST*10:     i++11:   **ELSE**12:     pop the *D(ST*[*sp*] off the *ST*.13:     *sp* = *sp*−*1*.14:   **ENDIF**15: **ENDWHILE**16: Output: *ST*.


Definition 4, Rotation direction of the path: Let *o* = *(x_o_,y_o_), p* = *(x_p_,y_p_), q* = *(x_q_,y_q_)* are any three nodes in the domain, vector *D(o,p,q)* denotes the rotation direction of the path.

$$D(o,p,q)=\left|\begin{array}{ccc} x_{o} & y_{o} & 1\\ x_{p} & y_{p} & 1\\ x_{q} & y_{q} & 1 \end{array}\right|=x_{o}y_{p}+y_{o}x_{q}+x_{p}y_{p}-x_{q}y_{p}-y_{q}x_{o}-x_{p}y_{o}$$

If *D* > 0, then the path <*o,p,q,o*> forms an anti-clockwise loop; if *D* < 0, then the path *(o,p,q,o)* forms a clockwise loop; if *D* = *0, o, p, q* are collinear.

We first identify the smallest *y* coordinate of nodes in *S*, assume *p*_0_ and establish a coordinate axis whose origin is *p*_0_ (if two nodes *p_i_* and *p_j_* have the same smallest *y* coordinate and *p_i_.x*<*p_j_.x*, we select *p_j_* as its origin). The other nodes are mapped to the *p*_0_ origin coordinate axis system. After mapping all of the nodes into the *p*_0_ origin coordinate axis system, we compute all of the node's slope and sort all of the nodes in ascending order according to the node's slope and get the sorted nodes set *T* = *{p*_1_,*p*_2_,*âĂę.p_n_}*, where *p*_1_ and *P_n_* have the smallest and largest slope, respectively.

**Figure 4.**
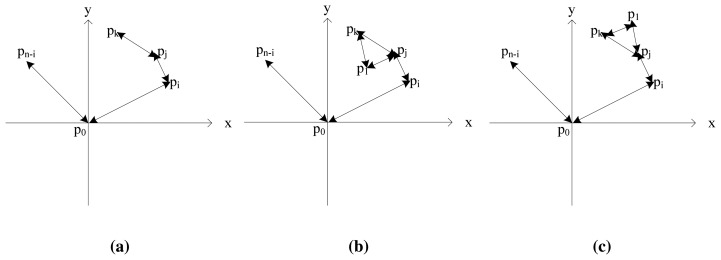
Finding the boundary sensors.

Second, we establish the stack *ST(S)*, which is initialized to *ST(S)* = *{P_n_, p*_0_*}*. Without loss of generality, We assume that at a time, the *ST(S)* = *{P_n_, p*_0_…*p_i_,p_j_,p_k_}*, where *p_k_* is on the top of *ST*, and the nodes in *ST* have constituted a semi-closed convex polygon (Figure 4a); *p_l_* is the next node in the *T*.

If the rotation direction *D (p_j_,p_k_,p_l_)* >0, then we push *p_l_* into stack *ST(S)*, since the path <*p_j_, p_k_, p_l_*> forms an anti-clockwise loop and <*p_j_, p_k_, p_l_*> forms a convex polygon, and the *p_k_, p_l_* are a convex polygon edge. If *D (p_j_,p_k_,xp_l_)* >0, then we pop *p_k_* out of stack *ST(S)* (Figure 4b,c), since the path <*p_j_, p_k_, p_l_*> forms an clockwise loop and *p_k_, p_l_* are not a convex polygon edge. Finally, the nodes in *ST* are the boundary sensors that determine the boundaries of a heritage object.

#### 3.3.2. Boundary Detection

##### Heritage Object Deformation Detecting by Checking the RSSI of Anchor Nodes

The wireless signal energy will decay with increasing distance in the process of communication. The signal energy received by the node is the RSSI. According to the log path loss model in [36], the received signal energy decay in a logarithmic trend with distance increases. If both the transmission energy and the received signal energy can be obtained at the receiving end, the signal attenuation can be obtained according to Formula (1) as follows:

(1) $$P_{r}(d)[dBm]=P_{0}(d_{0})-10n{\text{log}}_{10}(\frac{d}{d_{0}})$$

where *d*_0_ is the reference range, *P*_0_(*d*_0_) and *P_r_*(*d*) are the received signal strength in *d*_0_ and *d*, respectively, and *n* is the path loss exponent. We know from Formula (1) that the received signal strength of nodes is a function of the distance *d* and will be changed with the variation of *d*.

Transform Formula (1) to Formula (2):

(2) $$d=\left(10^{\frac{p_{0}(d_{0})[dBm]-p_{r}(d)[dBm]}{10}}\right)\times d_{0}$$

According to Formula (2), given *P_r_*(*d*), *d*_0_ and *P*_0_(*d*_0_), we can get the new reference range *d*, where the path loss exponent *n* is a fixed value and can be measured in an experiment if the environment is unchanged.

**Figure 5.**
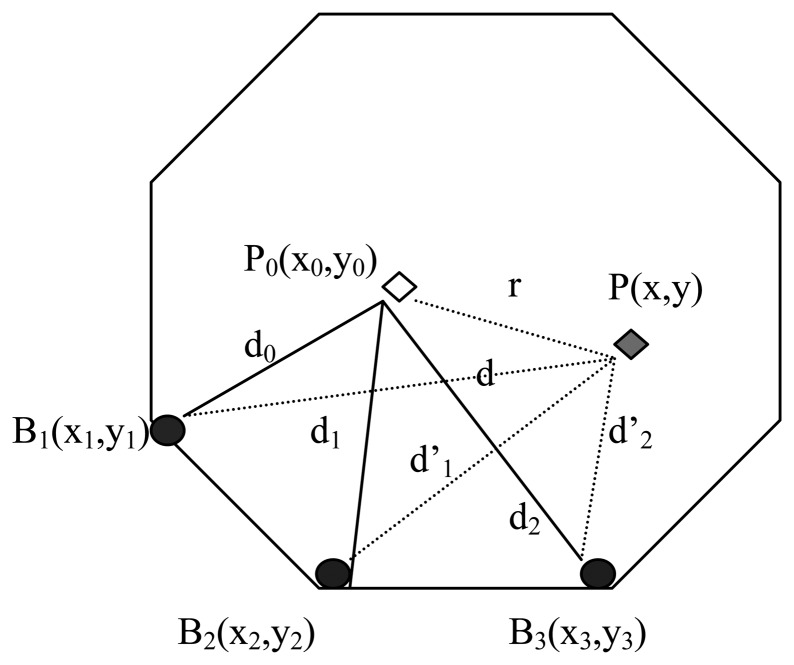
Changed position of the anchor nodes after deformation.

For example, In Figure 5, The new position of anchor node *P*_0_ is *P*, the distance between *P* and *B*_1_, *B*_2_, *B*_3_ is *d, d*_1_, *d*_2_, respectively, and the coordinate of *B*_1_, *B*_2_, *B*_3_ is (*x*_1_,*y*_1_)ïijŇ(*x_2_,y*_2_),(*x*_3_,*y*_3_), respectively. We can get the new position of *P′*s coordinate (x,y) according to Formula (3). By fusing the RSSI data of anchor nodes that are deployed on the heritage object, we can accurately calculate the heritage deformation size and angle. According to our actual experiment, the average positioning error is 0.115 m.

(3) $$\left\{\begin{array}{c} {\left(x-x_{1}\right)}^{2}+{\left(y-y_{1}\right)}^{2}=d^{2}\\ {\left(x-x_{2}\right)}^{2}+{\left(y-y_{2}\right)}^{2}=d_{1}^{2}\\ {\left(x-x_{3}\right)}^{2}+{\left(y-y_{3}\right)}^{2}=d_{2}^{2} \end{array}\right\}$$

We get *P′*s coordinate (x,y) by transforming Formula (3).

$$\left[\begin{array}{c} x\\ y \end{array}\right]={\left[\begin{array}{cc} 2(x_{1}-x_{3}) & 2(y_{1}-y_{3})\\ 2(x_{2}-x_{3}) & 2(y_{2}-y_{3}) \end{array}\right]}^{-1}\left[\begin{array}{c} x_{1}^{2}-x_{3}^{2}+y_{1}^{2}-y_{3}^{2}+d_{2}^{2}-d^{2}\\ x_{2}^{2}-x_{3}^{2}+y_{2}^{2}-y_{3}^{2}+d_{2}^{2}-d_{1}^{2} \end{array}\right]$$

In the actual environment, the signal attenuation model is influenced by the influencing factors, such as temperature, humidity, wind and other environmental factors, such as voltage and antenna. The signal attenuation model is not an ideal type as Formula (1), but in line with the normal distribution related to the distance [36], as shown in Formula (4).

(4) $$P_{r}(d)[dBm]=P_{0}(d_{0})-10n{\text{log}}_{10}\left(\frac{d}{d_{0}}\right)+X_{\sigma }$$

where *X_σ_* is the random variable in line with the normal distribution; *σ* is the noise factor under specific circumstances. When the environment is stable, the path loss exponent *n* and noise factor *σ* can be considered as a fixed value and can be obtained in an experiment. Therefore, *P_r_*(*d*) is a function of *d* and *X_σ_* and in line with the normal distribution. In order to improve the accuracy of calculation, we replace *P*_0_(*d*) and *P_r_*(*d*) with *E(P*_0_(*d*)*)* and *E(P_r_*(*d*)*)*, as well as replace *X_σ_* with *X̄_σ_*, where *E(P*_0_(*d*)*)* is the expected value of the received signal strength of *P*_0_(*d*), *E(P_r_*(*d*)*)* is the expected value of the received signal strength *P_r_*(*d*), *X̄_σ_* is the mean value of *X_σ_*. We can get Formula (5) from Formula (2).

(5) $$d=\left(10^{\frac{E(p_{0}(d_{0})[dBm])-E(p_{r}(d)[dBm])+{\bar{X}}_{\sigma }}{10n}}\right)\times d_{0}$$

##### Heritage Object Deformation Detecting by Boundary Detection of Heritage Site

Firstly, the DH finds the boundary of the domain. The DH (domain head) determines the sensors located at the boundary of the domain and notifies them to be the boundary sensors of the domain. The DH determines the boundary sensors among the normal nodes in the domain by the FBS algorithm in Algorithm 3. Note that hereafter, the boundary sensors of the domain are referred to as domain-boundary sensors (DBs), while the remaining nodes are referred to as normal sensors (Ns). As show in Figure 1, the shaded gray nodes represent the DBs of the corresponding domain.

The control messages are used to transmit the detecting information to the DH whenever the object is detected. There are “Detecting” and “Domain” in the control message. The format of “Detecting” and “Domain” is number. The “Detecting” implicate the Domain-boundary-sensors detect the target heritage, while “Domain” is the number of domain where the heritage is detected.

“Detecting”: is used only by the DBs and is sent to the DH once the DBs detect the target heritage. For example, when the DBs detect the collapse of the large heritage and parts of the heritage move into the domain, The DBs will set the “Detecting” to ‘1’ and send to DH.

“Domain”: is used only by the Ns. It is set to ‘*n*’ when the detected heritage is identified within domain *n*.

**Figure 6.**
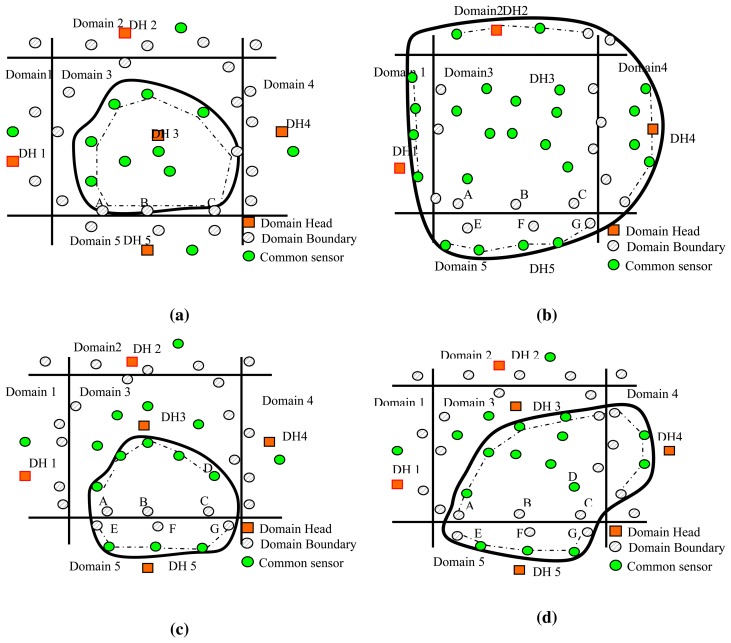
Boundary detecting and tracking of a heritage object. (**a**) The heritage object covers a single domain; (**b**) the heritage object shape fully covers a domain; (**c**) the heritage shape spread across two domains; (**d**) the heritage shape spread across three domains.

When the collapse or the deformation of the heritage object is detected, a DBs communicates with all of the one-hop neighboring DBs in other domains to query their detection information. Once the DBs has received this information, it sets “domain” to ‘*n*’ in the control message and sends it to the DH, such that the DH can determine all of the domains within which the heritage has spread. For example, we consider a network system comprised of just five domains, as shown in Figure 6. Two particular scenarios are presented in the following to explain how many domains the heritage object covers.

###### Scenario 1: Within a Single Domain or Covers the Whole Domain

The scenario in Figure 6a shows that the heritage object (indicated by the solid curve) is located entirely within a single domain (Domain 3). In this case, after sensing the heritage, the Ns within the object boundary broadcast ‘detecting’ messages to notify DH3 that they have detected the heritage object. Meanwhile, when the DBs in Domain 3 (*i.e.*, Sensors A, B, C) detect the heritage object, they query the object detection information of their one-hop neighboring DBs in Domain 4 and 5 and determine that the detected heritage does not extend beyond the boundary of Domain 3. The DBs set the ‘3’ to “domain” in the control message and send it to DH3. When receiving these messages, DH3 determines that the detected object is currently spread only within its own domain, DH3, uses FBS to distinguish the boundary sensors of the heritage, treats all of the sensors (including both the Ns and the DBs) as a subset Sand applies the FBS to get the corresponding boundary sensor set of the heritage object; for example, the boundary sensor set indicated by the dotted line in Figure 6a.

Let us consider the scenarios that the heritage object completely covers one or more than one domain. For example, as shown in Figure 6b, the heritage completely covers Domain 3. In this scenario, all of the DBs in Domain 3 detect the object and learn from their one-hop neighboring DBs that the scope of the detected heritage extends into the neighboring domains. Thus, once DH3 has received the “domain” messages from all of the DBs of Domain 3, it knows that its domain is completely covered. DH3 confirms that all of the nodes in Domain 3 are non-boundary sensors of the detected heritage object and therefore takes no further action. However, by inspecting the control message that they receive from their DBs, DH1, 2, 4, 5, *etc*. know that a portion of the heritage object boundary lies within their domain and, thus, employ the corresponding measures described in the case of “crosses multiple domains” to estimate its location.

###### Scenario 2: Crosses Multiple Domains

Figure 6c,d illustrates the scenario in which the heritage extends across two and three domains respectively; for the scenario in Figure 6c, the Ns in Domain 3 transmit “detecting” messages to DH3, as long as they detect the whole or parts of the heritage. Meanwhile, DBs A, B, and C query their one-hop neighboring DBs, *i.e.*, E, F and G, respectively; these sensors report that the object has indeed spread to Domain 5, and thus, DBs A, B and C set ‘3’ and ‘5’ to “domain” in the control message and then send it to DH3 (note that DBs D queries its neighbor in Domain 4). When DH3 receives the control messages, it learns that the detected heritage object is not confined solely within its own domain, but has also spread to Domain 5. As for the scenario in Figure 6d, DH3 learns that the detected heritage object is spread across three domains. If the heritage object crosses multiple domains, the domain head will estimate the portion of the boundary lying within its own domain, fuses the boundary information in a compact data format and then relays it to the sink via the connected core. The sink determines the entire boundary of the heritage site by compiling the integrated boundary information received from all of the domain heads in the network. The heritage object boundary detection (HBD) algorithm is presented in Algorithm 4.


**Algorithm 4** Heritage boundary detection (HBD) algorithm
1: **IF** the heritage within a single domain, **THEN**2: get the heritage object boundary by executing FBS.3: **ELSE**4: DHs estimates the portion of the object boundary lying within its own domain by executing the BPE (the boundary portion estimation algorithm (Algorithm 5)).5: All DHs fuse the boundary information in a compact data format and then relay it to the sink.6: The sink determines the entire boundary of the heritage by compiling the integrated boundary information received from all of the DHs in the network.7: **ENDIF**



**Algorithm 5** The boundary portion estimation algorithm (BPE)
**Step 1:** DHs distinguish the domain boundary sensors among all of the sensors that have detected the heritage object by FBS.**Step 2:** DHs identify and eliminate the redundant sensors in the domain boundary set.**Step 3:** DHs eliminate any non-boundary sensor(s) from the heritage object boundary set.


In Step 2, the DH identifies and eliminates the redundant sensors in the domain boundary set by eliminating the DBs that separate the distance into two sensor pairs. For example, in Figure 6c, DH3 calculates the straight line distance between each pair of DBs along the common border between Domains 3 and 5, which have detected the heritage object, *i.e.*, A and B, B and C and A and C; the distance between A and C is greater than that between A and B or B and C, respectively, and thus, B is eliminated. In Figure 6d, DH3 removes the redundant sensors, B and D.

In Step 3, on the basis of Step 2, DHs eliminate the redundant sensors that have the information that the shape of the detected heritage spreads across multiple domains. For example in Figure 6d, it is easily determined that node C cannot be a boundary sensor of the heritage object since it is not only a domain-boundary sensor in Domain 3, but also has the information that the shape of the detected heritage object spreads across more than two domains. Therefore, DH3 further eliminates Node *C* from the heritage object boundary set.

#### 3.3.3. Boundary Tracking and Deformation Detection

##### Boundary Tracking

After being deployed and initialized, EffeHDDT operates in the monitoring phase; the EffeHDDT method detects the heritage object boundary periodically. When a portion of or the whole heritage object boundary moves out of the sensing range of the current boundary sensors (for example, the deformation and collapse of the heritage object), on receiving these messages and examining the “domain” information they contain, DH identifies the nodes within its domain that represent the new boundary sensors of the heritage object by executing the heritage object boundary detection algorithm (Section 3.3.2), and the heritage object boundary node set must be updated to be responsible for the new boundary location. If a sensor detects the disappearance of the heritage object in its local area at the current time slot, it knows that the boundary of the heritage object moved through its detection area during the past time slot. In such a situation, the sensor set the “detection” to ‘−1’ in the control message to notify its DH of updating the heritage object boundary information.

**Figure 7.**
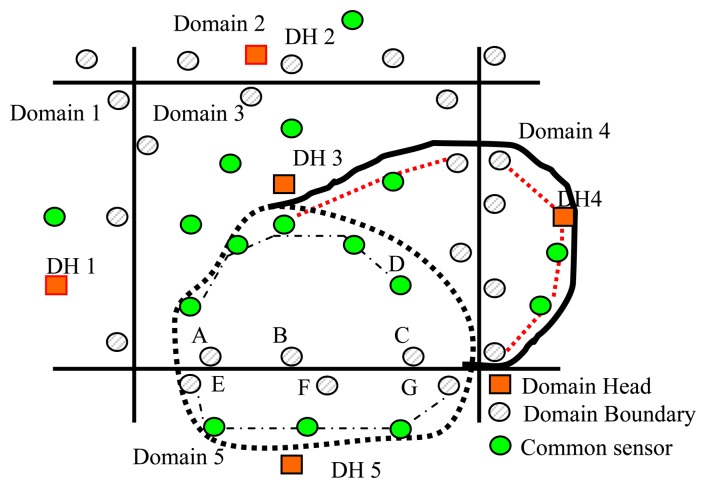
Updating boundary sensors when a heritage object is deformed or has a moving boundary.

Figure 7 illustrates a change in the position of the object boundary and the subsequent changes in the boundary profile portions in Domains 3, 4 and 5, respectively. Note that in these figures, the region via the thick dotted line represents the location of the heritage object in the previous time slot, and the circles connected via the thin dotted lines represent the old boundary sensors. Meanwhile, the curve marked using a thick solid line represents the new boundary. In the current time slot, the Ns and DBs in each domain sense the heritage object for the first time and send “detecting” and “domain” messages to their DH, and the EffeHDDT method identifies the new boundary sensors of the heritage object by executing the heritage object boundary detection algorithm (Section 3.3.2). The circles connected via red dashed lines in each static domain in Figure 7 indicate the new boundary sensors of the object, as determined by DHs 3, 4 and 5, respectively.

##### Deformation Detection

EffeHDDT detects the deformation of heritage object by the heritage object boundary similarity mechanism. The main idea is to save the latest heritage object boundary information OCP (old convex polygon) calculated in the last round in the DH or sink. In the new period, EffeHDDT will get the new heritage object boundary information NCP (new convex polygon), then calculate the similarity of the OCP and NCP (SM (C) Similarity). The heritage object deformed only when SM (C) is less than the threshold *K*(SM(*C*) < *K*) (in this paper, *K* = 0.93, 0.93 is given by the heritage experts [2]). At the same time, EffeHDDT broadcasts the threshold *K* to DHs and boundary nodes. DH and boundary nodes calculated the similarity and forwarded the new boundary information only when the similarity was less than *K*.

How does one calculate the convex polygon similarity? The two convex polygons are similar if they are topologically similar, geometrically similar, directionally similar and have the same area; the two convex polygons are topologically similar if they have a one to one corresponding relationship in the geometry element type and connection sequence; the two convex polygons are geometrically similar only if they are topologically similar and their connected type (vertical, tangent connection, *etc.*) of geometry element has a one to one corresponding relationship; the two convex polygons are directionally similar if they have the same angle between the minimum polygon rectangle and the horizontal axis; the two convex polygons are similar if they are topologically similar, geometrically similar, directionally similar and have the same area. The convex polygon similarity SM (*C*) can be defined as:

(6) $$SM(C)=K_{1}\times sm(\text{Top}\log y)+K_{2}\times sm(\text{Geometry})+K_{3}\times sm(\text{Direction})+K_{4}\times sm(\text{Area})$$

where *sm(Top log y), sm(Geometry), sm(Direction)* and *sm(Area)* are the topology similarity, geometry similarity, direction similarity and area similarity of the convex polygon, respectively. *K*_1_, *K*_2_, *K*_3_,*K*_4_ are the weight and *K*_1_ + *K*_2_ + *K*_3_ + *K*_4_ = 1. Similar to the image similarity calculation [37], we give the following general formula of *sm(T)*,

(7) $$sm(T)=\frac{\overset{M}{\underset{i=1}{\Sigma }}\overset{M}{\underset{j=1}{\Sigma }}\alpha_{ij}\beta_{ij}}{M\times N}$$

where *α_ij_* is the geometric elements of convex polygons and *β_ij_* is a similar coefficient of geometric elements [37].

## 4. Performance Evaluation

In this Section, we demonstrate the effectiveness and efficiency of EffeHDDT by a real node experiment and a simulation experiment.

### 4.1. Real Node Experiment

Typically, the preservation of cultural relics or heritage objects has higher requirements on the environment. This experiment is in a laboratory that stores precision instruments, which is very similar to the places where precious cultural relics or heritage object are saved. There is specialized equipment to maintain the indoor temperature, to keep wind and dust stable and to maintain dryness, and at the same time, there is special equipment to screen out strong electric and magnetic fields.

In the experiment, we use the MICAz node of Crossbow to collect the RSSI value and to create a sample database. Ten anchor nodes are deployed randomly in the 10 m × 10-m region, which is surrounded by 30 boundary nodes. The average of the three boundary nodes corresponds to one anchor node to calculate the RSSI value. In order to simulate random heritage object deformation, we move the position of the anchor node randomly. The distance of each movement is limited to 0.2 m, and it must be ensure that the anchor nodes cannot move out of the scope of the boundary nodes. We collect 1000 data at each distance; 60% of them are treated as the sample data for training, and the remaining 40% are treated as test data; the abnormal data are processed before the training samples.

The average weighted distance is calculated following Formula (6) acting as the estimation distance between boundary nodes and anchor nodes. We define distance error as follows: distance error = actual distance – estimation distance. The actual distance is the line measuring the distance between boundary nodes and anchor nodes.

**Figure 8.**
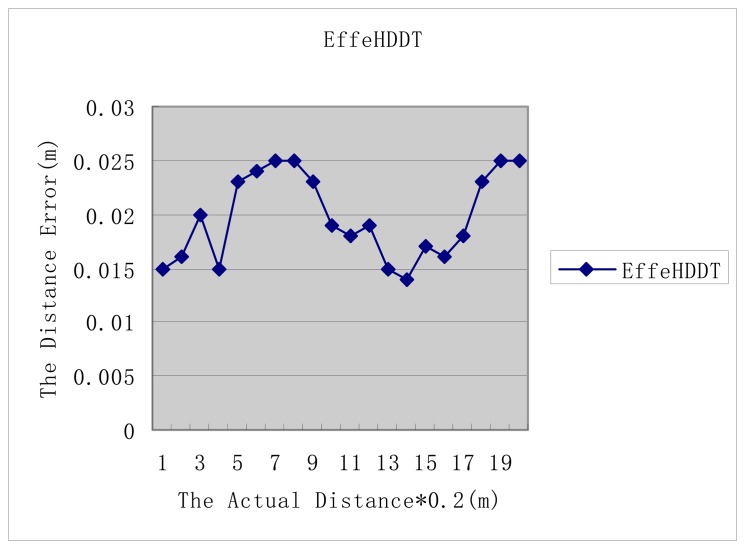
The distance error of EffeHDDT.

Figure 8 shows that the distance error results of EffeHDDT fluctuates between 0.015 to 0.025, which is a reasonable error range for a site deformation decision [2].

We carry out a positioning error experiment to test the positioning accuracy of EffeHDDT in the same experiment environment as the distance estimation experiment. If anchor node *P*'s coordinates in the *P_i_* position is (*x_i_, y_i_*) and was moved by *K* times, the final estimated position coordinate is ( $x_{i}^{\prime }$, $y_{i}^{\prime }$). The definition of positioning error γ for the anchor node is:

(8) $$\gamma =\frac{\overset{k}{\underset{i=1}{\text{∑}}}\left(\sqrt{{\left(x_{i}-{x^{\prime }}_{i}\right)}^{2}+{\left(y_{i}-{y^{\prime }}_{i}\right)}^{2}}\right)}{k}$$

**Figure 9.**
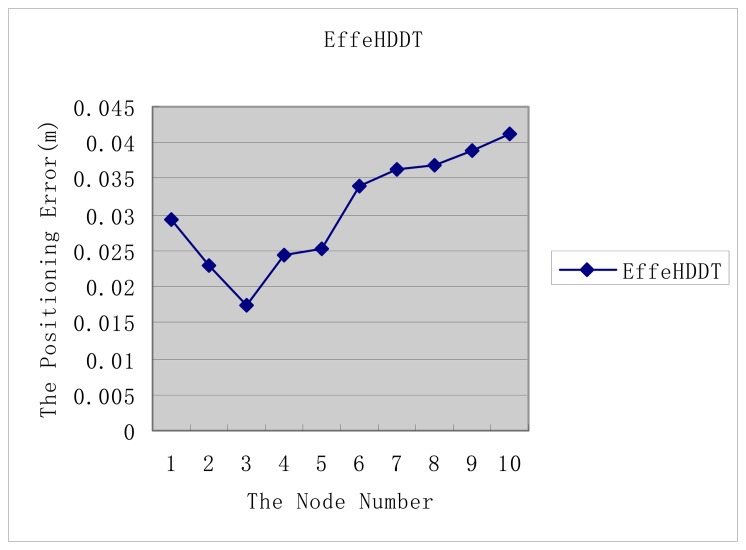
The positioning error of EffeHDDT.

Figure 9 shows that the positioning error results of EffeHDDT fluctuate between 0.018 to 0.042, which is a reasonable error range for a site deformation decision [2].

### 4.2. Simulation Experiment

#### 4.2. Simulation Experiment

We analyze the time efficiency of EffeHDDT by recording the average processing time for issuing a query to the network to obtain all of the results. All of the simulation experiments run in the network simulator 2 (ns2) [38].

We assume that the sensor network is randomly deployed in a simulated 1000 m × 1000-m sensing field. The radio range of each sensor was assumed to be 3 m. Two heritage sits/objects are simulated to be detected and tracked within the sensing field, a rectangle of 200 m × 100 m and a circle with a radius of 100 m, respectively. We assume that the rectangular and circular objects are initially centered at coordinates of (500, 600) and (200, 200), respectively. The sink is located at coordinates (0, 0). In order to simulate the deformation and collapse of the heritage object, the width and length of the rectangular heritage object and the radius of the circular heritage object increase by 0.1 m in each time slot, and the system generates a tension value *V* randomly at the same time, when the surface pressure exceeds *V*, the heritage object is split into multiple small parts and moves around with a random speed.

#### 4.2.1. Communication Cost

**Figure 10.**
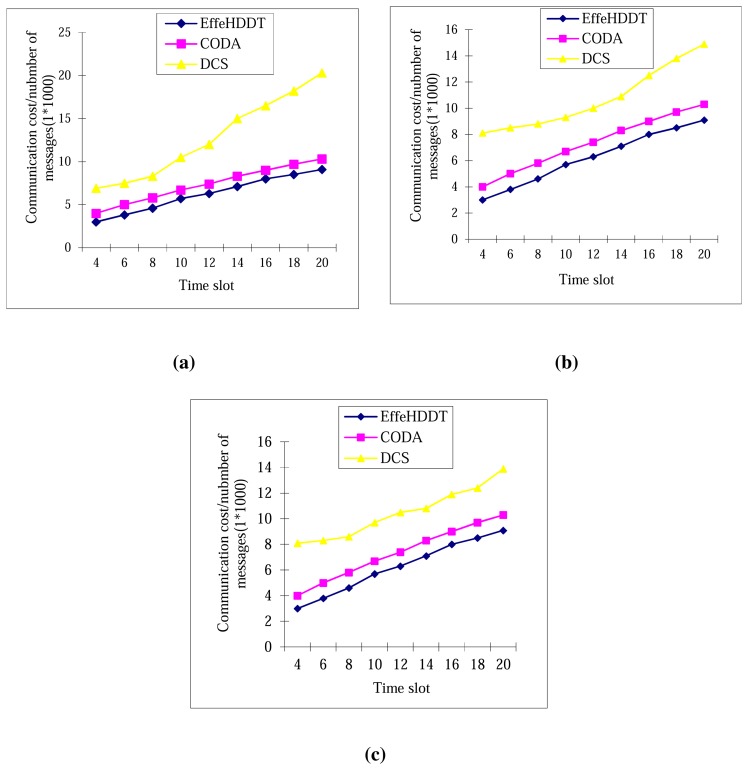
Communication costs over time (EffeHDDT with 50 domains and CODA (continuous object detection and tracking algorithm) with 50 clusters). (**a**) Dynamic clustering scheme (DCS) algorithm [18], with 50 boundary sensors; (**b**) DCS with 160 boundary sensors; (**c**) DCS with 220 boundary sensors.

**Figure 11.**
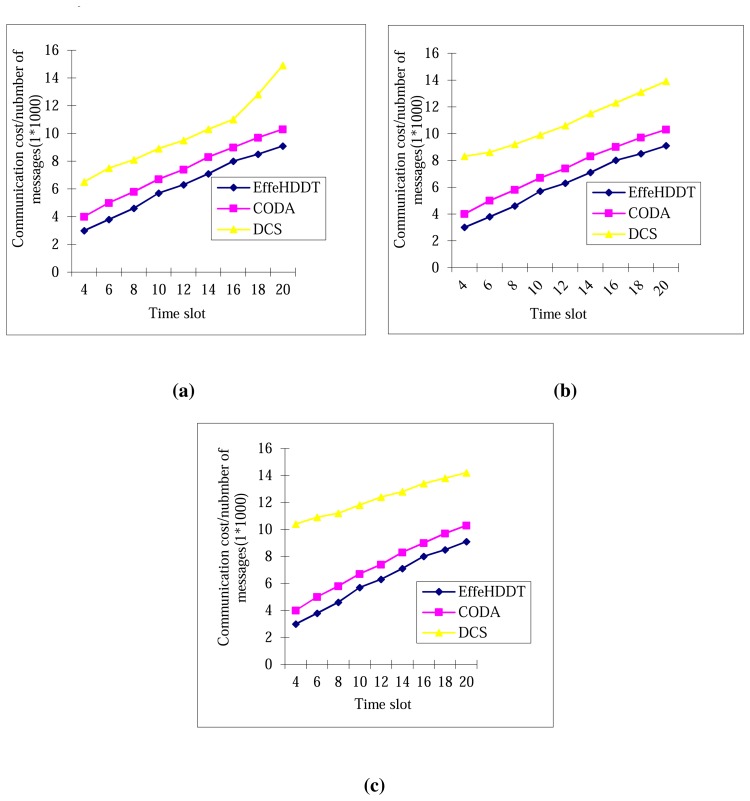
Communication costs over time (EffeHDDT with 50 domains and CODA with 50 clusters). (**a**) DCS with 50 dynamic clusters; (**b**) DCS with 100 dynamic clusters; (**c**) DCS with 150 dynamic clusters.

In this section, we analyze the communication costs incurred in detecting and tracking the boundary deformation and collapse of the heritage object. The communication cost is defined as the total number of data packets broadcast in establishing the boundary nodes, integrating the local boundary information and then disseminating this information to the sink. In the simulations of the message cost, the sensing field is assumed to contain a total of 2,500 sensors. We compared the communication cost of our EffeHDDT method with those of both CODA and DCS in three different boundary sizes in Figures 10 and 11. We first introduce the simulation setup. In Figure 10a–10c, we adopt a maximum number of 80, 160 and 220 boundary sensors with dynamic clusters in DCS, and 50 static and 50 domains in both CODA and EffeHDDT. In Figure 11a–11c, we adopt 50, 100 and 150 dynamic clusters without limitation of the size in each dynamic cluster in DCS, and 50 static clusters and 50 domains in both CODA and EffeHDDT. Then, comparing the three schemes, the overall trend in both Figures 10 and 11 is that EffeHDDT results in a considerably lower communication overhead, since EffeHDDT constructs the connected core and the DHs in the domains determine the boundary sensors of the two moving heritage objects. By contrast, in DCS and CODA, the boundary sensors are determined by requiring every sensor detecting the emergence of the object to communicate with all of its one-hop neighboring sensors. Dynamic clusters are then built by performing an extensive message exchange among all of the boundary sensors. Therefore, the total message costs of both DCS and CODA are very high.

**Figure 12.**
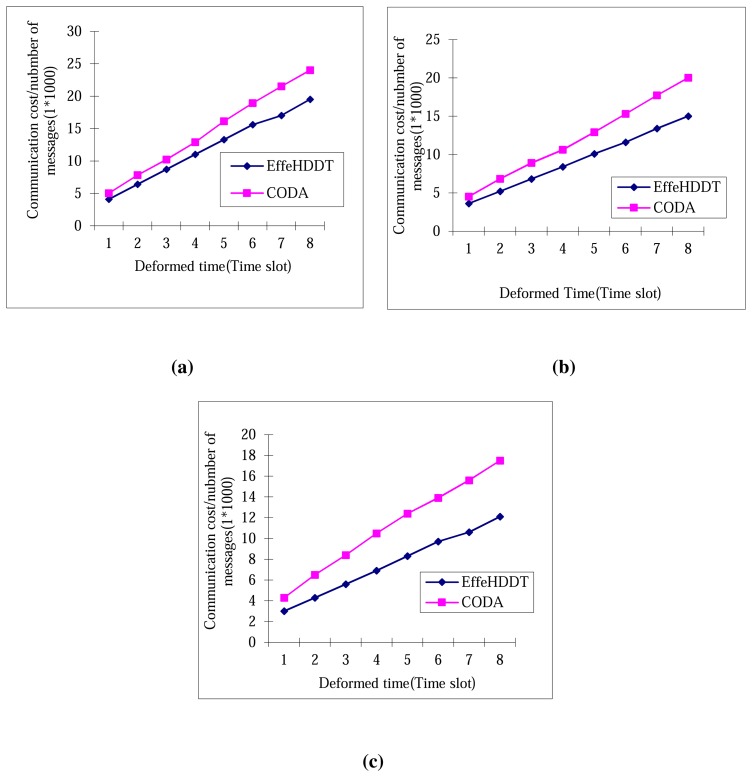
Communication cost over time with deformed speed. (**a**) EffeHDDT with 100 domains and CODA with 100 clusters; (**b**) EffeHDDT with 150 domains and CODA with 150 clusters; (c) EffeHDDT with 200 domains and CODA with 200 clusters.

The communication costs for control message broadcast changing with diffusion speeds are analyzed in Figure 12a–12c. The simulation setup is that we adopt 100, 150 and 200 static clusters in EffeHDDT and 100, 150 and 200 domains in CODA, and the width and length of the rectangular heritage object and the radius of the circle are increased by 0.1 m in each period of diffusion. It can be seen that the EffeHDDT has the least (best) communication cost. The reason is that when the control message in transmitted in the connected core in the EffeHDDT, the average communication cost occurs in domains less than the CODA when tracking diffused objects with a large size.

#### 4.2.2. The Precision of the Estimated Boundary

We define the precision of the estimated boundary as the boundary precision ratio (e/a) with a range of [0, 1]; that is, the actual boundary (a) is divided by the estimated boundary (e). We use the number of sensors within the actual heritage object boundary, representing the actual boundary, and the number of sensors enclosed by the estimated heritage object boundary as the estimated boundary. The precision of the estimated boundary is one if the number of sensors enclosed by the estimated heritage object boundary is equal to the number of sensors within the actual heritage object boundary. In fact, the number of sensors enclosed by the estimated heritage object boundary is less than the number of sensors within the actual heritage object boundary. Thus, the greater the value of the boundary precision ratio (e/a), the greater is the precision of the estimated boundary.

**Figure 13.**
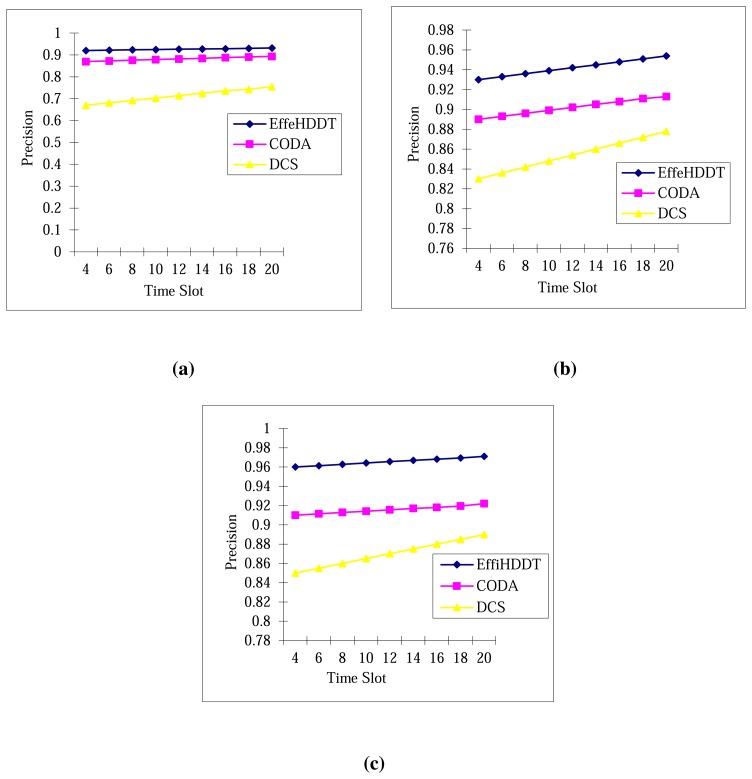
Boundary detection precisions changing with time: (**a**) 2000 nodes; (**b**) 2500 nodes; (**c** 3000 nodes.)

Figure 13a–13c compares the estimation boundary precision ratio of EffeHDDT, CODA and DCS in networks with three sensor densities (2000, 2500 and 3000 nodes). The common trend is that the three schemes retain an approximately constant estimation performance as the heritage size increases. For any given time slot, it can be seen that the heritage object boundary precision of the three schemes increases as the node density increases. Furthermore, we have observed that the EffeHDDT consistently outperforms both CODA and DCS, since EffeHDDT locates the boundary by finding the minimum convex polygon that contains the heritage object.

## 5. Conclusions

It is significant to monitor and signal the early warnings of the deformation of heritage objects. In this paper, we provide an EffeHDDT method). In EffeHDDT, we discover a connected core to form a back-bone path for transmitting and collecting control messages among the sensor nodes and, thus, reduce the communication cost greatly. Particularly, we develop a heritage object boundary detecting and tracking mechanism. Both theoretical analysis and experimental results demonstrate that EffeHDDT exceeds the existing work in terms of communication cost and precision of the estimated boundary.
